# Evaluation of a Prototype of a Novel Galactomannan Sandwich Assay Using the VIDAS^®^ Technology for the Diagnosis of Invasive Aspergillosis

**DOI:** 10.3389/fcimb.2021.669237

**Published:** 2021-07-16

**Authors:** Salomé Gallet, Cécile Garnaud, Céline Dragonetti, Sophie Rivoiron, Odile Cognet, Yuping Guo, Mylène Lesénéchal, Danièle Maubon, Muriel Cornet

**Affiliations:** ^1^ Laboratoire de Parasitologie-Mycologie, Département des Agents Infectieux, Institut de Biologie et de Pathologie, CHU Grenoble Alpes, Grenoble, France; ^2^ Univ. Grenoble Alpes, CNRS, CHU Grenoble Alpes, Grenoble INP, TIMC, Grenoble, France; ^3^ Department of Immunoassays, R&D bioMérieux, Marcy l’Etoile, France

**Keywords:** diagnosis, invasive aspergillosis, galactomannan, single-sample test, VIDAS^®^

## Abstract

**Objectives:**

To evaluate the analytical and clinical performance of a prototype of a VIDAS^®^ Galactomannan (GM) unitary test (bioMérieux, Marcy l’Etoile, France) and compare to that of the Platelia™ *Aspergillus* Ag assay (Bio-Rad, CA, USA).

**Methods:**

Repeatability, reproducibility, and freeze-thaw stability of VIDAS^®^GM were evaluated. Sera from patients at risk of IA were concurrently tested with both the VIDAS^®^GM and Platelia™ *Aspergillus* Ag assays. Correlations between the two assays were assessed by Passing Bablok (PB) regression and performance by ROC analysis.

**Results:**

The correlations between the VIDAS^®^GM indexes after one and two cycles of freezing/thawing were r=1.00 and r=0.989, respectively. The coefficients of variation for negative, low-positive, and positive sera were 13%, 6%, and 5% for repeatability and 14.4%, 7.2%, and 5.5% for reproducibility. Overall, 126 sera were tested with both assays (44 fresh and 82 frozen). The correlation between VIDAS^®^GM and Platelia™ *Aspergillus* Ag was r=0.798. The areas under the curve of the ROC analyses were 0.892 and 0.894, for VIDAS^®^GM and Platelia™ *Aspergillus* Ag, respectively.

**Conclusions:**

This new VIDAS^®^GM prototype assay showed adequate analytical and clinical performance and a good correlation with that of Platelia™ *Aspergillus* Ag with 126 sera, although these results need to be confirmed in a larger prospective and multicentric study. As for the other VIDAS^®^ assays, VIDAS^®^GM is a single-sample automated test using a solid reagent strip and receptacle. It is easy to use and suitable for rapid on-demand test results.

## Introduction

Invasive aspergillosis (IA) is an opportunistic infection that occurs mainly among immunocompromised patients. Its incidence has increased with the increasing use of immunosuppressive therapies (Vallabhaneni et al., 2017). IA is associated with high morbidity and mortality, especially if diagnosis and treatment are delayed (Cornely et al., 2017). Since 1990, biological markers, mainly galactomannan (GM), have considerably improved IA diagnosis and its precocity (Guo et al., 2010; Jenks et al., 2019). Until very recently, Platelia™ *Aspergillus* Ag (BioRad), based on a sandwich enzyme immuno-assay (EIA) technique, was the most widely used for GM detection. A meta-analysis showed a pooled sensitivity and specificity for proven cases of 0.71 and 0.89, respectively (Pfeiffer et al., 2006). However, well-known limitations include poor reproducibility and repeatability and the need to batch the samples in series, resulting in a loss of speed (Oren et al., 2012). Several lateral-flow devices assays for detecting either GM or another antigen have been recently commercialized to respond to the need of a rapid and easy-to-use single-sample test (Thornton, 2008; Jenks and Hoenigl, 2020; Mercier et al., 2020). These tests performed better on broncho-alveolar lavages than sera, in which the sensitivity was lower than that of Platelia™ *Aspergillus* Ag (Donnelly et al., 2020). Here, we evaluated the analytical and clinical performance of a prototype of a VIDAS^®^GM (BioMérieux) unitary test. As for the other VIDAS tests, it is a fluorescent EIA packaged in ready-to-use disposable strips.

## Methods and Results

This monocentric retrospective and prospective study included 126 sera from 30 patients at risk of IA at the University Hospital of Grenoble (France). The patients had mainly hematological malignancies (n=27) including allogeneic bone marrow (n=21) and solid organ transplantation. Eighteen probable and 6 possible IA were diagnosed by a local multidisciplinary Aspergillosis committee. The EORTC/MSG criteria were used to classify the patients (Donnelly et al., 2020). Probable cases were mostly classified as such on the basis of the GM (Platelia™ *Aspergillus* Ag) results and/or *Aspergillus* PCR. The possible cases (only radiological/clinical criteria) were not considered as IA cases. Patients were screened for GM detection with Platelia™ *Aspergillus* Ag and the samples collected as part of routine clinical care and registered in the certified biological collection DC-2008-582. Both frozen and fresh samples were analyzed. Frozen and fresh samples were tested the same day with the two assays to assess the effect of storage at -80°C on GM detection. Platelia™ *Aspergillus* Ag was performed according to the manufacturer’s instructions using an automated EVOLIS Premium^®^ system (BioRad) and a GM index cut-off value of 1 was considered for positive samples as recommended in the recent revision of the EORTC/MSG criteria (Donnelly et al., 2020). VIDAS^®^ GM is an automated qualitative sandwich assay with a coated solid-phase receptacle that also serves as a pipetting device. Samples are heat pre-treated with EDTA, as for the Platelia™ *Aspergillus* procedure. In the instrument, after a dilution step, GM is captured between the coated mouse monoclonal antibody (mAb) and the detection rat mAb conjugated to biotin. Alkaline phosphatase linked to an anti-biotin antibody hydrolyzes the substrate into a fluorescent product at 450 nm. The assay prototype uses a standard (S1) and a positive control. A relative fluorescence value (RFV) is generated and automatically calculated by the instrument, according to S1, and an index value (I) is calculated as I=RFVsample/RFVS1. IA cases were classified as proven or probable according to the 2020 EORTC/MSG criteria (Donnelly et al., 2020). Appropriate permissions have been obtained from BioMérieux for the copyright of the VIDAS^®^ trademark.

**Figure 1:**
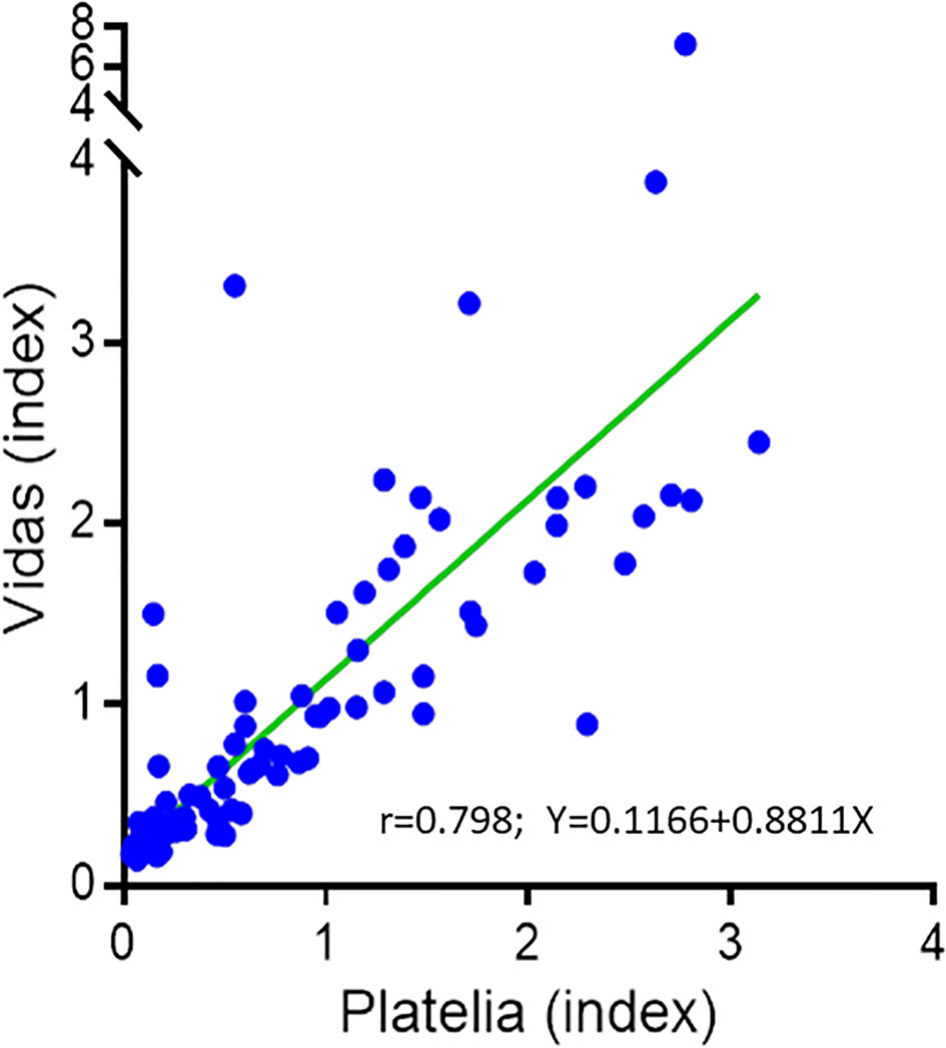
Correlation between VIDAS^®^GM and Platelia™ *Aspergillus* Ag using serum samples. N = 118 (42 fresh and 76 frozen, samples with Platelia results ≥ 3.5 were excluded).

**Figure 2:**
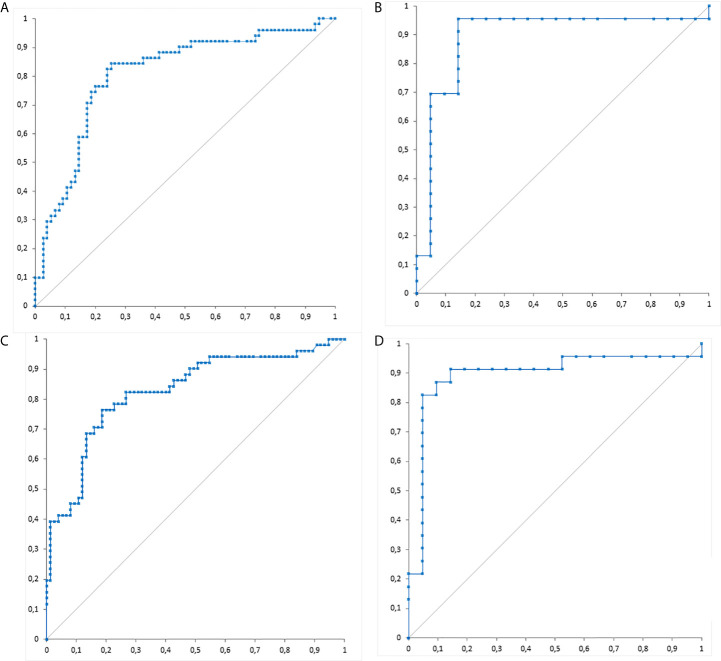
Receiver operating characteristic (ROC) curves of VIDAS^®^GM for **(A)** total serum samples and **(B)** serum samples collected 15 days before or after the date of invasive aspergillosis diagnosis. ROC curves of Platelia™ *Aspergillus* Ag for **(C)** total serum samples and **(D)** serum samples collected 15 days before or after the date of invasive aspergillosis diagnosis.

Overall, 126 sera were tested with both assays (44 fresh and 82 archived at -80°C). We evaluated the stability of VIDAS^®^GM after one and two cycles of freezing after seven days (at -80°C) and thawing (at room temperature) using 9 and 11 samples, respectively. We used the Passing Bablok (PB) test and analyse-it 5.0 software. The PB showed excellent correlations between the VIDAS^®^GM indices after one (r=1.00) and two cycles (r=0.989) of freezing/thawing. The repeatability (precision within run) and reproducibility (total precision) of VIDAS^®^GM were evaluated from four sera (one negative, one low-positive, and two positive) measured in triplicate twice a day for 3 days, totalizing 18 measurements per sera. The coefficients of variation for negative, low-positive, and positive sera were 13%, 6%, and 5% and 14.4%, 7.2%, and 5.5%, respectively (SAS Add-in 9.2 software). The PB correlation between VIDAS^®^GM and Platelia™ *Aspergillus* Ag levels was r=0.798 (**Figure 1**). There was a good agreement, with a Cohen kappa index of 0.82 (95% CI=0.71-0.93) between the two assays for the positive and negative results based on a VIDAS^®^GM cut-off of 1, calculated from the PB equation (**Figure 1**; Y=0.1166+0.8811X), and a 1 Platelia™ *Aspergillus* Ag cut-off. The performance of the assays assessed by ROC curves is shown in **Figure 2**. Considering all 126 sera, the areas under the curve (AUC) were 0.808 and 0.827 for the VIDAS^®^GM and Platelia assays, respectively. For the sera collected 15 days before or after the date of the IA diagnosis AUC of the two assays were better and similar (0.892 and 0.894, respectively). The cut-offs and results that correspond to the higher Youden index (the best balance between sensitivity and specificity) for both tests are shown in **Table 1**. A cut-off of 0.36 for the VIDAS^®^GM assay can thus be proposed based on these ROC analyses and Youden index.

**Table 1:** Clinical performance of the VIDAS^®^GM and the Platelia™ *Aspergillus* Ag assays using ROC analyses and best Youden index and serum samples of patients at risk for invasive aspergillosis.

	Sensitivity	Specificity	PPV*	NPV*	AUC*	Youden	Cut-off
All sera (n = 126)							
VIDAS^®^GM	0.843	0.747	0.694	0.875	0.808	0.590	0.36
Platelia™ Aspergillus Ag							
cut-off close to 0.5	0.765	0.813	0.736	0.836	0.827	0.578	0.47
cut-off close to 1	0.471	0.880	0.730	0.710		0.350	1.01
Sera collected 15 days before or after the IA diagnosis (n = 44)							
VIDAS^®^GM	0.957	0.857	0.880	0.947	0.892	0.814	0.36
Platelia™ Aspergillus Ag							
cut-off close to 0.5	0.870	0.905	0.909	0.864	0.894	0.774	0.55
cut-off close to 1	0.609	0.952	0.930	0.690		0.561	0.97

*PPV, positive predictive value; NPV, negative predictive value; AUC, area under the curve.

The median VIDAS^®^GM and Platelia™ *Aspergillus* Ag index levels of the IA cases were 0.98 and 0.88 for IA cases and 0.26 and 0.125 for non-IA patients, respectively.

## Discussion

GM detection has been used for IA diagnosis since the early 90’s. The EIA Platelia™ *Aspergillus* Ag kit rapidly supplanted the latex agglutination Pastorex A*spergillus* ^®^ kit (BioRad), which was commercialized first. Newly developed single-sample tests have addressed the need to reduce the time to results for early-targeted therapy and an improved outcome of IA patients (Thornton, 2008; Jenks and Hoenigl, 2020; Mercier et al., 2020). Here, we evaluated a prototype of a novel single-sample GM assay, VIDAS^®^GM and compared it to Platelia™ *Aspergillus* Ag. VIDAS^®^GM showed excellent stability, repeatability, and reproducibility. The correlation between the two assays was also high (r=0.798) and their diagnostic performance comparable, with the AUC under ROC curves of 0.892 and 0.894 for the VIDAS^®^GM and Platelia assays, respectively (**Figure 2**). In **Figure 1** showing the correlation the three points with low indices of Platelia™ *Aspergillus* Ag and high indices of the VIDAS GM correspond to false negative of the Platelia in probable IA patients (these 3 patients presented other sera positive with the Platelia™ *Aspergillus* Ag). Importantly, the IA diagnosis was established according to the revised EORTC/MSG criteria (Donnelly et al., 2020), which include GM itself. Thus, the results of the diagnostic performance of the two assays should be interpreted with caution, as the sensitivity may have been overestimated. Nevertheless, the IA diagnosis remains possible when excluding GM from the diagnostic criteria, as our patients fulfilled the risk factors, as well as the clinical and radiological EORTC/MSG features.

The ROC curves and best Youden index revealed a VIDAS^®^GM cut-off of 0.36, corresponding to a sensitivity of 0.957, a specificity of 0.857, and an AUC of 0.892 when selecting the sera surrounding the IA diagnosis. This cut-off needs to be confirmed in further larger studies.

The new VIDAS^®^GM single-sample assay provides a semi-quantitative measurement of GM, a widely used biomarker for which biologists and physicians have developed substantial expertise in interpreting. During the course of infection and treatment, fluctuations of the level of the VIDAS^®^GM indexes were consistent with those of Platelia™ *Aspergillus* Ag and the patient’s outcome (data not shown). The main benefit of VIDAS^®^GM is that it is a simple ready-to use system adapted for VIDAS instruments, thus providing rapid results (70 min).

This novel GM single-sample assay showed suitable analytical (stability, repeatability, reproducibility) and clinical performance and good correlation with that of the Platelia™ *Aspergillus* Ag assay. These results need to be confirmed in a larger prospective, multicentric study in which the diagnosis may be defined by composite criteria without GM. Such future studies will allow refinement of the cut-off for sera and the analysis of respiratory samples.

## Data Availability Statement

The raw data supporting the conclusions of this article will be made available by the authors, without undue reservation.

## Ethics Statement

The samples collected in this study are part of routine clinical care and registered in the certified biological collection DC-2008-582. This collection is approved by the ethical committee of the Centre Hospitalier Universitaire of Grenoble. The patients/participants provided their written informed consent to participate in this study.

## Author Contributions

All authors listed have made a substantial, direct and intellectual contribution to the work, and approved it for publication.

## Conflict of Interest

CD, SR, YG, and ML are bioMérieux employees.

The remaining authors declare that the research was conducted in the absence of any commercial or financial relationships that could be construed as a potential conflict of interest.

The reviewer FG declared a past co-authorship with several of the authors DM, MC, to the handling Editor.
